# Correspondence regarding 'Clouded leopards, the secretive top-carnivore of South-East Asian rainforests: their distribution, status and conservation needs in Sabah, Malaysia'

**DOI:** 10.1186/1472-6785-7-5

**Published:** 2007-07-02

**Authors:** Chris H Gordon, Anne-Marie E Stewart, Erik Meijaard

**Affiliations:** 1 The Nature Conservancy, East Kalimantan Program, Jalan Gamelan 4, Samarinda 75123, East Kalimantan, Indonesia

## Abstract

Correspondence regarding Wilting A, Fischer F, Bakar SA, Linsenmair KE: **Clouded leopards, the secretive top-carnivore of South-East Asian rainforests: their distribution, status and conservation needs in Sabah, Malaysia**. BMC Ecol 2006, **6**:16.

Wilting *et al*. have provided their response to Gordon *et al*., directly following the correspondence.

## Background

Due to their secretive behaviour, nocturnal habits and low densities, there has been a distinct lack of research conducted on clouded leopards (*Neofelis *spp.), and thus little information exists on their ecology, distribution and behaviour. Clouded leopards probably spend a large amount of their waking hours moving on the ground, during both day and night [1], and have been recorded using logging roads for travel [2-4], with Gordon and Stewart [4] noting that logging roads do not act as ecological barriers to clouded leopard territories. Furthermore, clouded leopards have even been recorded using logging roads for the purposes of hunting, and for marking their territory [4]. Their use of roads offers one of the few opportunities to observe signs of clouded leopards without using the expensive techniques of camera trapping or radio-collaring.

Most studies on large solitary felids apply radio telemetry and camera trapping to estimate home range sizes and densities [5-7]. However, in a recent paper, Wilting *et al*. [2] attempt to estimate the population size of Sundaic clouded leopards (recently renamed as *Neofelis diardi*) [8] in their study area through the identification of individuals from their tracks. They then proceed to extrapolate this information to estimate the distribution and status of the clouded leopard in the whole of Sabah, Malaysian Borneo. Below, we address four issues raised by Wilting *et al*. that we consider to fall short of scientific standards.

## Critique of Wilting *et al*

1. Most previous work on individual recognition of felids through their tracks has concentrated on tigers (*Panthera tigris*) [9-12], although other studies have looked at mountain lion (*Felis concolor*) [13-15] and snow leopard (*Uncia uncia*) [9]. Karanth *et al*. [10] determined that 30 years of pugmark censuses to estimate tiger abundance in India failed because statistical assumptions for abundance estimates were not considered and even with 30 years of censuses, data on spatial distribution was still lacking. While Wilting *et al*. [2] adopted more rigorous methods, we maintain that the recognition of individual felids from pugmark sets is extremely difficult. Their principal component analysis (PCA) of tracks does indeed separate the six track sets into three or four groups, but this method is flawed because each of the 50 tracks in the six track sets is treated as an individual case, whereas a PCA assumes that cases are independent. It is therefore not surprising that the individual tracks group so clearly; they were part of a dependent set of tracks made by one individual. Wilting *et al*. should have averaged track measurements for each track set and conducted their PCA with six rather than 50 cases. This reduced sample size is too small to reliably extrapolate to a large area.

2. When investigating population sizes and space use of carnivores, metabolic requirements and energy acquisition, as influenced by prey availability, are also important aspects to take into account [16,17]. Prey diversity and abundance are features that vary between sites, for example logged and unlogged forests, or areas where hunting is permitted [18-20] and these features will certainly have an effect on the population of clouded leopards that can be supported in such areas. However, Witling *et al*. [2] have given no consideration to the effect of prey base when estimating clouded leopard population sizes, and have extrapolated information obtained in one site with a particular prey assemblage to other forest areas throughout Sabah. The close proximity of their study areato oil palm plantations and to a river would affect local prey densities and consequently could result in higher clouded leopard densities. Mammal densities derived from such locations are liabletooverestimation, and mistakes at the smaller scale will become more pronounced when extrapolating these numbers to forested areas across the entire state.

3. The main threat to clouded leopards apart from forest loss is hunting [16]. Although hunting pressure on particular species in Borneo remains mostly unstudied, it varies considerably. Hunting in coastal, Muslim-dominated areas focuses on different species than in Borneo's interior. Also, local culture and food preferences determine whether certain species are targeted [[21], TNC unpubl. data]. Without knowing how hunting pressure varies within Sabah, extrapolation of densities from one protected area to the whole of the State is unjustified.

4. Wilting *et al*. [2] conclude that there are 8–17 individuals per 100 km^2 ^in their study area. While the authors maintain that their density estimate is just that, an estimate, and make the point of referring to it as a rough figure, the variance in their estimate is considerable, especially as they have attempted to calculate populations for the whole Sabah region. They assume that all forested areas would have similar densities. In doing so, they have also ignored small isolated populations that fall outside larger protected areas and reserves. It has been shown that clouded leopards are present in areas of heavy logging and high human activity, with suggestions that home range size may be larger in such habitats [4] and consequently, densities lower.

As a result of the above factors, the available data do not justify the estimation of the number of clouded leopards in the study area and, even less the extrapolation, however careful, of such data regarding the size of populations in an entire region, especially when spatial variation in threats such as hunting, or ecological requirements, have not been quantified.

## Conclusion

We appreciate Wilting *et al*.'s [2] attempt to further knowledge on a poorly known species, which, as one of the top predators in Borneo, likely plays an important ecological role. We disagree, however, with their far-reaching conclusions regarding population densities and regional estimates, which find little support in the data provided. Furthermore, the authors claim that they have found a method of studying even secretive cats in tropical rainforests using thorough quantitative track surveys to identify individuals. Yet they had no independent method to check their track-based conclusions. Track surveys in tropical forest areas are a quick and inexpensive technique to determine the presence of a certain species [22] and could assist with the decision of where to place cameras or cage traps. However, when estimating total population size, this method should be used in conjunction with the more reliable and proven techniques of camera trapping and radio-collaring.

While Wilting *et al*. [2] point out that they are the first to provide population estimates of clouded leopards in Sabah, this estimate is in danger of becoming a "quoted fact". In fact, this is already happening. Recent global media attention to the taxonomic upgrade of the Sundaic clouded leopard (*N. diardi*) to species level was accompanied by population estimates similar to, and possibly based on those by Wilting *et al*. [23].

In conservation planning it often happens that with a lack of reliable data, any available data are used to determine conservation priorities, especially when original sources are no longer consulted. Misguided information can be a powerful factor in guiding conservation policy and funding away from where it is most needed. Conservation scientists should therefore ensure that they provide reliable data, and if these are not available, refrain from making quantitative statements on the status or population trends of species. With that in mind, we find it important to point out some of the methodological weaknesses in Wilting *et al*.'s work, allowing those less familiar with the species or survey methodologies to put their conclusions in perspective.

## List of Abbreviations

TNC = *The Nature Conservancy*

## Authors' contributions

All authors contributed equally to this rebuttal. All authors read and approved the final manuscript.

## Response from original authors

Andreas Wilting, Frauke Fischer, K. Eduard Linsenmair

Address (University of Würzburg, Biocentre, Department of Animal Ecology and Tropical Biology 97074 Würzburg, Germany)

Corresponding author Andreas Wilting

Phone: +49-931-888 4316

Fax: +49-931-888 4352

Email: a.wilting@gmx.de

In our recent paper [2] we proposed the potential of a rigorous track classification method to study secretive carnivores in tropical rainforests. On the basis of six clouded leopard track sets we estimated a rough minimum density of clouded leopards in our small study area and extrapolated our local results to the landscape level.

We are grateful for the critical response by Gordon *et al*., but would like to emphasise, that we are fully aware of the limitations of the track classification method. Our extrapolated clouded leopard numbers were rather intended to be a first working hypothesis for further research than a reliable estimation of the actual population size in the whole State of Sabah.

We would like to respond to the main concerns of Gordon *et al*. to clarify our results and help to prevent misinterpretations that might have negative effects on the management of one of the most threatened cat species in Asia.

Correctly Gordon *et al*. pointed out, that it is extremely difficult to recognise individual felids from their tracks. Therefore we noted the limitations that have to be considered when applying the track classification method. The authors criticised that we applied a principal component analysis (PCA) to separate the track sets, although the tracks of the same clouded leopard were not independent. We disagree that the PCA assumes that the cases are independent, because we used the PCA only as a means of exploratory data analysis to reduce our original 14 variables to a two dimensional graph for better illustration. Because we did not statistically test our data e.g. by calculating the confidence intervals, our tracks do not have to be independent. Furthermore, the assignment of the tracks to the six track sets was part of the analysis. Gordon et al. suggested averaging track measurements for each track set. Applying this reduction we would have eliminated the variance of tracks within one track set. Further, within the scatter plots the track sets could never intersect with each other in space, because each track set would be only represented by one point in the graph. In summary we think that we have applied the PCA correctly and we feel safe to presume a minimum number of four clouded leopards in our study site.  

We are aware that it can never be guaranteed by pugmark assessment to track all individuals in a study area and fully agree with Gordon et al. that due to hard and unsuitable substrates the capture probabilities of an individual might be lower than using, for example, a camera-trapping approach. We tried to overcome these uncertainties by applying a capture-recapture analysis, which incorporates the capture probabilities. However, due to the low number of recaptures in our study we emphasized that our calculated density should rather be taken as a rough minimum estimate and not as the true number. Nevertheless, we suppose, fully in line with previous methodological publications [e. g. [9,11][13-15]], that the track classification method will have a high potential for further research activities; presumably not to provide true numbers, but rather as a cheap alternative to estimate rough minimum numbers in a particular site. We totally agree with Gordon et al. that the calculated track-based estimates have to be checked by an independent method like camera-trapping, and we are currently planning to apply both methods in different study sites for such an evaluation.  

Furthermore, Gordon et al. criticized that we up-scaled our local results to the landscape level. We are equally concerned and well aware of the fact that without any detailed information about the other areas such extrapolations are based on very weak evidence. We discussed the problems resulting from this approach in our publication (different legal hunting and poaching pressures; different forest structures and protection status of the reserves, and different prey abundances in the reserves). The authors are right that the close proximity of our study site to the delimitated oil palm plantation affect the density of potential prey species, but without any information about the extent of regional differences and without any knowledge about clouded leopards’ preferred prey species in Borneo, we were not able to consider metabolic requirements in our rough calculation. 

Gordon *et al*. are right to criticised that we ignored the smaller isolated populations. However, we did not intend to give an estimation of the total numbers of clouded leopards in Sabah, we rather wanted to locate areas that might be large enough to hold a viable population (defined as > 50 individuals). Therefore, we excluded those smaller populations on purpose. Being aware of all these uncertainties in our rough estimation we still suppose that as a first working hypothesis these figures are of great value for future research. It is a first tentative step to fill a tremendous gap of knowledge. For a species with such limited information on its distribution and status, even very rough estimates, based on limited data, are valuable and important. Gordon et al. are right in stating that these numbers should not become a “quoted fact” in literature. They should rather motivate researches to test these numbers during intensive field studies and help to set priorities for future research plans. We are equally concerned about the “high” population estimates stated in the global media for Borneo and Sumatra. However, we neither gave any estimates for the whole of Borneo nor for Sumatra. If the numbers in the global media are based on our rough estimates for Sabah the discussion in our paper was not sufficiently considered and for the best of this species we hope that more precise data will be provided by further research very soon.  

These upcoming research activities are of even greater importance, because recent reclassification of clouded leopards suggests a distinct species (N. diardi) on the Sundaland islands Borneo and Sumatra [8][24,25]. Furthermore a wider genetic sampling by Wilting et al. [25] indicates limited gene flow and population division between the islands of Borneo and Sumatra. This reclassification puts the distinct Sundaland clouded leopards on Borneo at an even greater risk of extinction.
